# The Year-Book of Treatment for 1892

**Published:** 1892-03

**Authors:** 


*The Year-Book of Treatment for* 1892. Pp. 486. London: CASSELL & COMPANY, LIMITED.

This present number of *The Year-Book of Treatment* gives a very complete summary of the most important papers on therapeutics published during the past year, and thus forms a trustworthy book of reference to the recent advances in treatment in surgery, medicine, and the other branches of our art. We may say of the book as a whole that the abstracts are uniformly well done, judiciously selected, and arranged in convenient form for ready reference. It is hardly necessary to say more of the individual sections than that all the writers give a satisfactory account of the year's work in their respective subjects. As might be anticipated, the accounts of the clinical experiments made with Koch's tuberculin take up a good deal of space in many of the sections. The immense amount of work that has been done in investigating the value of the remedy here becomes apparent. No treatment of recent times has received so thorough a trial in all quarters of the globe, and, we may add, few, if any, have so completely been found wanting. After the sections on the treatment of diseases, follows a summary of the new therapeutic remedies introduced during 1890-91, a selected list of new books and of new editions—English, French, German, and Italian,—and last, but not least, a full index to authors' names and to subjects.

The book may be cordially recommended as fully maintaining the reputation that the former issues have gained.

